# Molecular modeling of swine influenza A/H1N1, Spanish H1N1, and avian H5N1 flu N1 neuraminidases bound to Tamiflu and Relenza

**DOI:** 10.1371/currents.RRN1015

**Published:** 2011-01-03

**Authors:** Ly Le, Eric Lee, Klaus Schulten, Thanh N. Truong

**Affiliations:** ^*^Department of Chemistry, University of Utah; ^†^Beckman Institute for Advanced Science and Technology and ^§^Professor of Chemistry, University of Utah, & Director of ICST, Vietnam

## Abstract

A molecular model of the swine influenza A/H1N1 ( also called H1N1pdm) type-I neuraminidase was built using the pathogenic avian H5N1 type-I neuraminidase as a basis, due to the higher sequence identity between A/H1N1 and H5N1 (91.47%) compared to Spanish H1N1 (88.37%) neuraminidase. All-atom molecular dynamics (MD) simulations of all three neuraminidases were performed, either as apo-structures or with commercial antiviral drugs Tamiflu or Relenza separately bound; the simulations allowed for the identification of both conserved and unique drug-protein interactions across all three proteins. Specifically, conserved networks of hydrogen bonds stabilizing the drugs in the sialic acid binding site of the simulated neuraminidases are analyzed, providing insight into how disruption due to mutations may lead to increased drug resistance. In addition, a possible mechanism through which the residue 294 mutation acquires drug resistance is proposed by mapping the mutation site onto an electrostatic pathway which may play a role in controlling drug access to the binding pocket of neuraminidase, establishing a starting point for further investigations of neuraminidase drug resistance.

## Introduction

The three pathogenic viruses, the 1918 Spanish flu (H1N1), 2003 avian flu (H5N1) and recently the 2009 swine flu (A/H1N1), share the same subtype 1 (N1) of neuraminidase which is a glycoprotein component on a flu virus’ surface that cleaves the alpha-ketosidic linkage of sialic acid (SA) located on human cells to release new virions which then infect nearby healthy cells [1] . The two FDA antiviral drugs including oseltamivir (Tamiflu)  [2] and zanamivir (Relenza) [3] function as neuraminidase inhibitors that mimic the scaffold of SA.

To date, the reported cases of the 2009 swine A/H1N1 virus appear to indicate that this variant is less virulent than the avian H5N1 virus responsible for the 2003 outbreak.  However, the alarming rate at which A/H1N1 has spread throughout the world, due to human-to-human transmission, reminds one of the potential consequences should a virulent strain emerge.  Strong evidence exists that the A/H1N1 strain derived from human or avian influenza A viruses and includes a reassortment of the internal genes between different swine flu viruses [4].  The familial relationship between emerging strains of flu capable of infecting humans must be taken into account for vaccine and antiviral drug development. 

The atomic structure of A/H1N1 neuraminidase currently is not known.  However, the fact that the A/H1N1 strain responds positively to Tamiflu and Relenza [5], which are effective on H5N1, suggests similarities to H5N1 neuraminidase.  Here we present results from molecular dynamics (MD) simulations of an atomic model we have built for the A/H1N1 type-I neuraminidase along with H1N1 and H5N1 neuraminidases in apo-form and with Tamiflu and Relenza individually bound to each protein.  Our study focuses on characterizing the structural and chemical properties of the drug binding pocket and on the specific drug-protein interactions which are essential for drug binding.  Insights gained here increase our degree of preparedness against the A/H1N1 flu outbreak, which may yet evolve into a deadly pandemic.

## Material and Methods

### Molecular model of A/H1N1 neuraminidase

The amino acid sequence of A/H1N1 neuraminidase obtained from Genbank  Locus ID CY041156 and of H5N1/H1N1 neuraminidases from the Protein Data Bank. The sequence alignment performed using Multiseq in VMD [6] showed that A/H1N1 has a higher percent of sequence identity (91.47 %) with H5N1 than it has with H1N1 (88.37%).  Therefore, a molecular model of A/H1N1 was built using H5N1 as the starting point by mutating corresponding residues to match the wild type H1N1.  At the drug binding pocket, the notable difference between N1 and N5 neuraminidase is the replacement of Y347 by N347 

### Preparation of Starting Structures

The coordinates for neuraminidases of H1N1 bound with Relenza, H5N1 bound with Tamiflu, and H5N1 bound with Relenza were taken from Protein Data Bank (PDB) structures 3B7E [7], 2HU4 [8], and 3CKZ [9], respectively. The residue T274 in 3CKZ was mutated back to H as in wild type. The position for Tamiflu bound to H1N1 were adopted from its corresponding location in H5N1, as the two proteins’ binding pockets differ only by residue 347, located on a loop at the periphery of the active site.  Simulated systems without drug bound were taken from the bound structures with the drug deleted.  In total, nine systems were considered involving three proteins (H1N1, H5N1, and A/H1N1) either unbound, or bound to Tamiflu or Relenza.  The unbound simulations are labeled simH1, simH5, and simSW, respectively, with Tamiflu and Relenza bound simulations are labeled simH1-t, simH5-t, simSW-t, and simH1-r, simH5-r, and simSW-r, respectively as shown in Table 1. A schematic view of each simulation system is shown in Figure 1. 

**Table:** 

Name	Structure	Atoms	Ensemble	Time (ns)
simH1	H1N1	32778	NpT	20
simH5	H5N1	33543	NpT	20
simSW	A/H1N1	40112	NpT	20
simH1-t	H1N1 + Tamiflu	32801	NpT	20
simH5-t	H5N1 + Tamiflu	33527	NpT	20
simSW-t	A/H1N1 + Tamiflu	38020	NpT	20
simH1-r	H1N1 + Relenza	32800	NpT	20
simH5-r	H5N1 + Relenza	33458	NpT	20
simSW-r	A/H1N1 + Relenza	33463	NpT	20

Table1: Summary of simulations. The “ensemble” column lists the variables held constant during the simulations; N, p and T correspond to number of atoms, pressure, and temperature, respectively.

### Parameter Generation for Inhibitors

The CHARMM31 force field force field [10] for proteins with CMAP correction[11] was used for the simulations. Parameters for ligands were prepared using Paratool [12] in VMD[6]. The initial structures of Tamiflu and Relenza were taken from crystal structures of H5N1 neuraminidase with these drugs bound. Structure optimization and frequency calculations were performed at the level of HF/6-31G* and then imported into Paratool. Atom types and charges were manually assigned.  All atom types and their atomic charge were found in the available CHARMM force field [10], except for the atomic charges of some atoms in the six member rings whose interactions with other atoms were not yet parameterized.  The drug molecules were divided into several small fragments. The atomic charges for the six-member ring atoms were recalculated based on the total charge of each fragment. Fragments not explicitly defined in the CHARMM force field were modeled using analogs in CHARMM force field extensions which fit closely to the fragment being parameterized. The dihedral angles, which are highly rigid due to electron delocalizations, were generated from the quantum mechanical calculations. 

### Molecular Dynamics Simulations

The protein complexes, with and without drug bound, were solvated in a box of TIP3P[13] water (water box shown in Figure 1A) and ionized by NaCl (0.304 M) to mimic physiological conditions.  All simulations were performed using NAMD 2.6 [14] and the CHARMM31 force field with CMAP correction [10] [11].

The ionized systems were minimized for 10,000 integration steps and equilibrated for 20ns with 1 fs time steps.  Constant temperature (T = 300 K) was enforced using Langevin dynamics with a damping coefficient of 1 ps−1.  Constant pressure (p = 1 atm) was enforced using the Nosé-Hoover Langevin piston method. Van der Waals interactions cutoff distances were set at 12 Å (smooth switching function beginning at 10 Å) and long-range electrostatic forces were computed using the particle-mesh Ewald (PME) summation method.  Electrostatic maps were calculated using the APBS plugin of VMD.  Hydrogen bond analysis utilized a distance and angle cutoff of 3.5 Å and 60 degrees, respectively.

**Figure:**
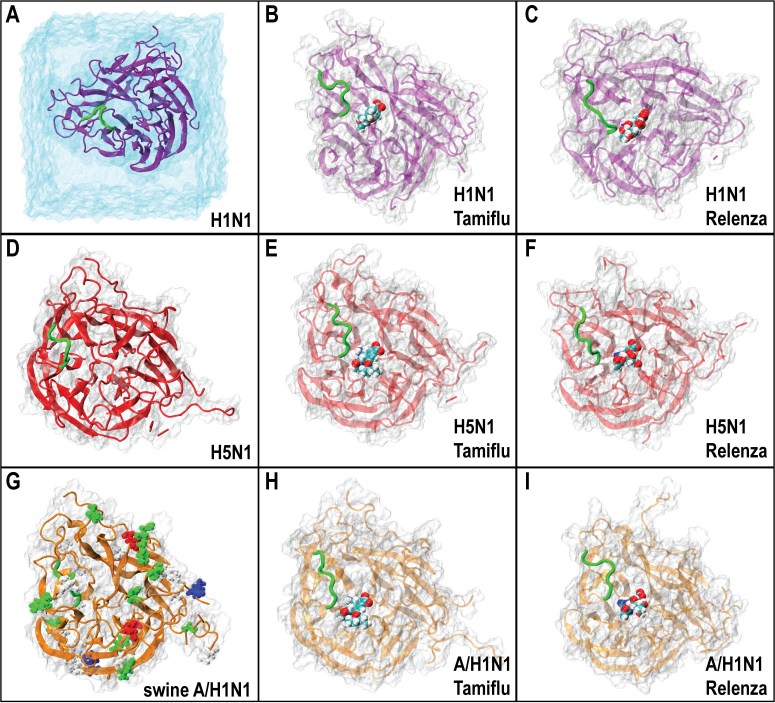

Figure 1. Illustrated table of nine simulated systems involving 1918 Spanish H1N1, Avian H5N1, and Swine A/H1N1 type 1 neuraminidases.  In A) the H1N1 protein is shown an explicit water box, the same setup being used in all nine simulations.  A critical loop (called 150) near the binding pocket believed to function as a laterally gate for drug binding, is shown in green for all figures.  In A-C), H1N1 is shown with no drug bound, Tamiflu bound, and Relenza bound, respectively.  The same is shown for D-F and G-I for H5N1 and A/H1N1 neuraminidases, respectively.  In G), the residues by which A/H1N1 swine flu neuradminidase differs from H5N1 avian flu neuraminidases are also shown to reflect the mutations made to the H5N1 structure to generate the model for A/H1N1 structure used in simulations. 

## Results and Discussion  

### Overall drug stability

Equilibrium simulations were carried out long enough for the drug-protein interactions to stabilize. The antiviral drugs Tamiflu and Relenza bind stably during simulation in the SA binding pocket of H1N1, H5N1, and A/H1N1.  The root mean squared deviations (RMSD) of the drugs are plotted over each simulation (Figure 2) and show that the position of the drugs remain fairly constant with minimal deviation within the binding pocket, permitting the characterization of the specific drug-protein interactions responsible for binding Tamiflu and Relenza tightly to the active site of neuraminidase type I.  The strong coupling between drug and protein simulated here may reflect the fact that the clinical efficacy of these drugs remains high for these wild type strains [5].   In simH1-t, a small shift in the RMSD for Tamiflu arose from the rotation of the bulky pentyl group. Loop 150 (shown in green for all panes of Figure 1) was observed to be rather flexible in apo simulations (simH1, simH5, and simSW), but relatively immobile for the drug bound simulations. This observation supports the important role of loop 150 proposed in an earlier study [15]. In addition, we found that adding calcium ions and treating crystal waters explicitly have minor effects on the final equilibrated structure.  The results will be reported in our formal publication.

**Figure:**
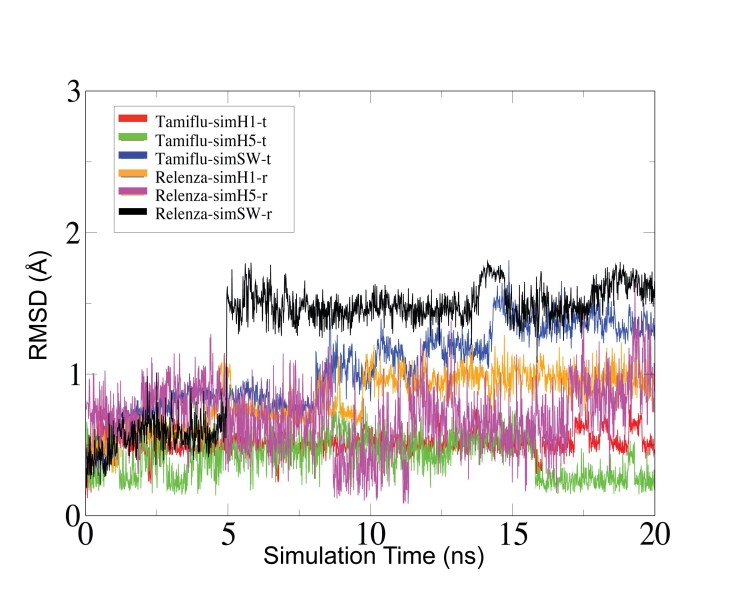

Figure 2: The antiviral drugs Tamiflu and Relenza bind stably during simulation in the SA binding pocket of H1N1, H5N1, and H1N1A.  Shown are the root mean squared deviations (RMSD) of the drugs for each simulation.  The values show that the positions of the drugs remain fairly constant with minimal deviation within the binding pocket, thereby permitting the characterization of the specific drug-protein interactions responsible for binding Tamiflu and Relenza tightly to the active site of neuraminidase type I.  

### Identifying conserved hydrogen bonds stabilizing Tamiflu and Relenza in the active sites of H1N1, H5N1, and A/H1N1

Hydrogen bonds play a key role for the ability of drugs to bind to influenza neuraminidases [16]. The hydrogen bonds forming between Tamiflu or Relenza and the residues lining the SA binding pocket of H1N1, H5N1, and A/H1N1 were determined over all drug-bound simulation trajectories.  The results of this calculation are shown in Figure 3, which depicts both schematic views of the residues involved in hydrogen bonding stably with the drug, and also histograms listing hydrogen bond frequency.   The hydrogen bonds observed to form in simH5-t and simH5-r agree well with previously published studies [8] [17] .  Specifically in simH5-t, we observe a high degree of conservation for hydrogen bonds between Tamiflu and residues E119, D151, R152, R292, and R371; in simH5-r, residues R118, E119, E227, E277, and R371 form hydrogen bonds with Relenza present throughout the duration of the simulation.  In the case of simH1-t and simH1-r, residues E119, E227, E27 and R371 form conserved hydrogen bonds with Tamiflu, and residues E119, E227, E276, E277, and R371 form conserved hydrogen bonds with Relenza, respectively.  In simSW-t and simSW-r, residues E119, D151, E277, R292, and R371 form conserved hydrogen bonds with Tamiflu, and residues R118, E119, E227, E277, E292, and E371 form conserved hydrogen bonds with Relenza, respectively.

**Figure:**
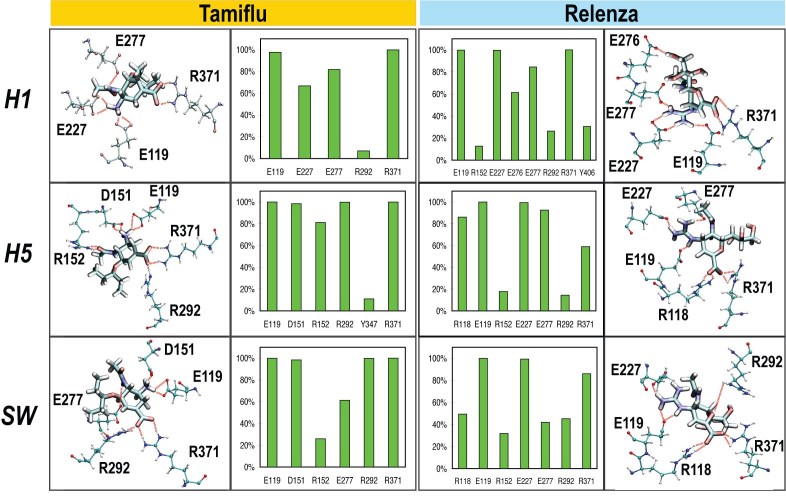

Figure 3: Network and occupancy of hydrogen bonds stabilizing the antiviral drugs osaltamivir (Tamiflu) and zanamivir (Relenza) in the sialic acid binding pocket of H1N1, H5N1, and H1N1A "swine" flu neuraminidase proteins.  Shown on the outer columns are schematic views depicting the protein residues which form conserved hydrogen bonds (shown as red dashed line) with Tamiflu (left side) and Relenza (right side).  The inner columns contain the corresponding histograms illustrating the occupancy (over 20ns of simulation time, see Methods) of each set of drug-to-sidechain hydrogen bonds.  Hydrogen bonds between Tamiflu and E119 and R371 were conserved across all three proteins; the same holds for hydrogen bonds between Relenza and E119, E227, and R371. Mutations disrupting these conserved hydrogen bonds may induce drug resistance. 

In all drug bound simulations, hydrogen bonds between Tamiflu and E119 and R371 were conserved across all three proteins while hydrogen bonds between Relenza and E119, E227, and R371 were conserved across all three proteins.   Specifically, R371 was always observed to hydrogen bond frequently with the carboxylate moiety of both Tamiflu and Relenza.  E119 hydrogen bonds with amino (NH_3_^+^) and guanidino (NHC(=NH_2_^+^)NH_2_) groups on Tamiflu and Relenza, respectively.   The guanidino group of Relenza also hydrogen bonds frequently with E227. Mutations disrupting these conserved hydrogen bonds may represent one mechanism of drug resistance.

Residue Y347, the only different residue between the SA active site of H5N1 and A/H1N1 neuraminidases, locates near a triarginyl cluster (Arg118, Arg292, Arg371) and is believed to form stabilizing hydrogen bonds with the carboxyl group of Tamiflu when bound to H5N1 [8] [17]. This same set of hydrogen bonds were seen initially in our simulation (during the first 2.5 ns), but were not conserved through the 20 ns trajectory, as the Tamiflu’s carboxyl group interacts very strongly and preferentially forms hydrogen bonds with R371.  Overall, the occupancy of the Tamiflu-Y347 hydrogen bond was less than 11% in our simulations, which sampled longer than an earlier study [17].  It was reported that the R292K mutation causes Tamiflu-resistance in neuraminidase type 2 (N2) which has N347 [8] [18]; the mechanism for this drug resistance is speculated to be that N347 does not interact strongly with Tamiflu’s carboxyl group, thereby leaving residue 292 as the lone hydrogen bond partner for stabilizing the protein interaction with the drug’s carboxyl group.  A mutation to residue 292 was expected to destabilize the drug-protein interaction and lead to drug resistance.   Our simulations do not support this speculation in the case of N1, due to our observation that the carboxyl group predominately forms hydrogen bonds with R371.   Further studies are clearly needed to contrast and/or connect the many mechanisms of drug resistance between the various types of flu neuraminidases.  

### The surface electrostatic potential of the SA binding pocket and implication of the drug resistance in H5N1

The electrostatic potential was mapped onto the surface of the protein, revealing that the SA pocket is actually rather negatively charged. Figure 4A shows an image of the electrostatic potential mapped onto the surface of H5N1 with Tamiflu bound.  Previous studies have identified that an oxocarbonium cation is formed as an intermediate during the process in which SA is cleaved by N1 neuraminidases.   The negative potential of the SA binding pocket may help to stabilize the oxocarbonium cation intermediate making this cleavage possible [19] [20] .  The simulations reveal, however, that the entrance to the SA pocket is ringed by a rather positive electrostatic potential, with the exception of a narrow path of negative potential leading into the binding pocket.  Figure 4C shows a closeup view of the SA binding pocket for all nine simulations, clearly illustrating this negatively charged pathway as it passes through the positively charged outer ring of the pocket. It turns out that residue 294, whose mutation has been correlated with drug resistance, is located along this path (shown in Figure 4B and at the head of green arrow in Figure 4C); this suggests that this negatively charge electrostatic surface pathway may play a key role for drug access into the SA binding pocket.   While not located directly on this negative electrostatic pathway, residue 274, which has been strongly correlated to Tamiflu resistance, is actually positioned adjacent to another residue, 292, which is located along this negatively charged pathway as well.  One can speculate that mutations to 274 may therefore indirectly impact the conformation or electrostatic potential of this narrow pathway into the binding pocket.

Previous studies investigating Tamiflu-resistance of H5N1 neuraminidase as a result of H274Y and N294S mutations have produced conflicting speculations on the mechanisms behind N1 drug resistance.  One study suggested that the H247Y mutation alters the conformation of the hydrophobic pocket within the SA binding pocket near Tamiflu’s ethyl moiety [16] while another study suggested that the mutation disrupts the E276-R224 interaction [21].  It has also been proposed that the N294S mutation may induce a side chain rotation of residue Y347 that leads to Tamiflu-resistance [21]. However, Tamiflu’s preferential binding to R371 instead of Y347 in our simulations suggest that a different mechanism may be at play here involving the electrostatic funnel on which residue 294 maps directly onto (Figure 4B). For A/H1N1 neuraminidase which has N347, the effect of N294S mutation remains unclear. Further studies are needed for a full understanding of N1 based drug resistance.

**Figure:**
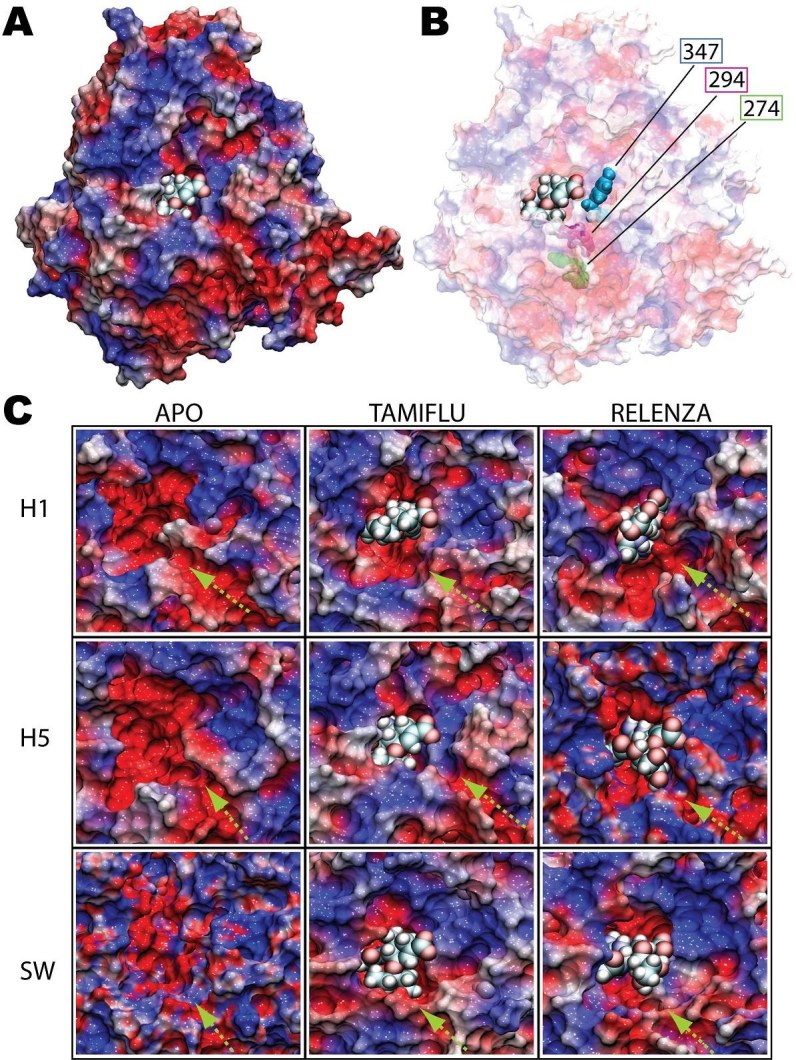

Figure 4:  Electrostatic surface potential of H1N1 (H1), H5N1 (H5), and A/H1N1 (SW) in drug-bound and unbound simulations.  Shown in A) is a stereoscopic surface view, colored by electrostatic potential, of H5N1 avian flu neuraminidase with Tamiflu bound at the SA binding pocket.  The region of the binding pocket where the drug binds has a highly negative potential (colored red) whereas the opening of the pocket is surrounded with highly positive potential ring (colored blue).   An exception is a negatively charged path leading into the binding pocket in B, the positions of residues 274, 294, and 347 are mapped onto the protein surface. A charged pathway (shown by the green arrow in the close-up views in C) was observed to be conserved in all nine simulations for all three proteins with or without drug bound.  The head of the green arrow corresponds to the location of residue 294 (also shown in B), whose mutation is a source of Tamiflu resistance.

## Conclusion and Suggestions

The World Health Organization has raised the A/H1N1 pandemic alert level to 6, its highest level.  A thorough understanding of this type of virus in all aspects (transmission, protection, vaccine, drug binding) is therefore important to increase our degree of preparedness. Besides continued surveillance of influenza in humans and animals, designing inhibitors effective over wide range of neuraminidase N1 subtypes and their mutants will provide the best possible preparation for a likely outbreak.  For this purpose, insight into the differences in binding of known drugs to the therapeutic targets will be useful.  A quantitative study of drug binding to A/H1N1, H5N1, and H1N1 neuraminidases and its mutant variants possessing drug resistance, will be crucial for revealing the precise mechanism of drug resistance. The study presented here not only presents the atomic level investigation of A/H1N1 neuraminidase, but also opens the door for future studies on the dynamics of drug binding/unbinding processes for all related N1 flu neuraminidases. 

## Acknowledgments

The authors thank Dr. J. Saam, Dr. H. Freedman, and Dr. R. Rizzo for their useful inputs.

## Funding information

This work is supported by grant(s) from the National Institutes of Health P41-RR05969. The authors gladly acknowledge the University of Utah's Center for High Performance Computing and an allocation at the Texas Advanced Computing Center via  Large Resources Allocation Committee grant MCA93S028. L. Le was partially supported by the Vietnam Education Foundation. 

## Competing interests

The authors have declared that no competing interests exist.  
